# Supplementary material for: Development of an Anti-HER2 Single-Chain Variable Antibody Fragment Construct for High-Yield Soluble Expression in Escherichia coli and One-Step Chromatographic Purification
Source: Biomolecules. 2023 Oct 11;13(10):1508. doi: 10.3390/biom13101508 (PMC10605039; doi:10.3390/biom13101508)

Supplementary Materials for

Article

Development of an Anti-HER2 Single-Chain Variable Antibody Fragment Construct for High-Yield Soluble Expression in *Escherichia coli* and One-Step Chromatographic Purification

Kyu Tae Byun [1,†], Boram Kim [1,†], Junmin Cho [1], Inbeom Lee [1], Myung Gu Lee [2], Dongsun Park [3], Tae-Bong Kang [1,4], Hyung-Sik Won [1,4] and Chan Gil Kim [1,*]

[1] Department of Biotechnology, Research Institute (RIBHS), College of Biomedical and Health Science, Konkuk University, Chungju, Chungbuk 27478, Republic of Korea
[2] Konkukbio Inc., Konkuk University, Chungju, Chungbuk 27478, Republic of Korea
[3] Department of Biology Education, Korea National University of Education, Cheongju, Chungbuk 28173, Republic of Korea
[4] BK21 Project Team, Department of Applied Life Science, Graduate School, Konkuk University, Chungju, Chungbuk 27478, Republic of Korea
* Correspondence: changil.kim@kku.ac.kr (C.K.)
† These authors contributed equally to this work.

Abstract: Although single-chain variable fragment (scFv) is recognized as a highly versatile scaffold of recombinant antibody fragment molecules, its overexpression in *Escherichia coli* often leads to formation of inclusion bodies. To address this issue, we devised and tested four different constructs, named v21 to v24, for producing anti-human epidermal growth factor receptor 2 (HER2) scFv. Among them, the v24 construct obtained from N-terminal fusion of maltose-binding protein (MBP) and subsequent tabacco etch virus protease (TEV) was identified as the most efficient construct for production of anti-HER2 scFv. Aided by the MBP tag, high-yield soluble expression was ensured and soluble scFv was liberated in cells via autonomous proteolytic cleavage by endogenously expressed TEV. The isolated scFv containing a C-terminal hexahistidine tag could be purified through a one-step purification via nickel-affinity chromatography. The purified scFv exhibited a strong (nanomolar K_d) affinity to HER2 both *in vitro* and in cells. Structural and functional stabilities of the scFv during storage for more than one month were also assured. Given the great utility of anti-HER2 scFv as a basic platform for developing therapeutic and diagnostic agents for cancers, the v24 construct and methods presented in this study are expected to provide a better manufacturing system for producing anti-HER2 scFv with various industrial applications.

Keywords: antibody; single-chain variable fragment (scFv); human epidermal growth factor receptor 2 (HER2), bacterial production; *Escherichia coli*; maltose-binding protein (MBP); tabacco etch virus (TEV) protease

Supplementary Figure S1

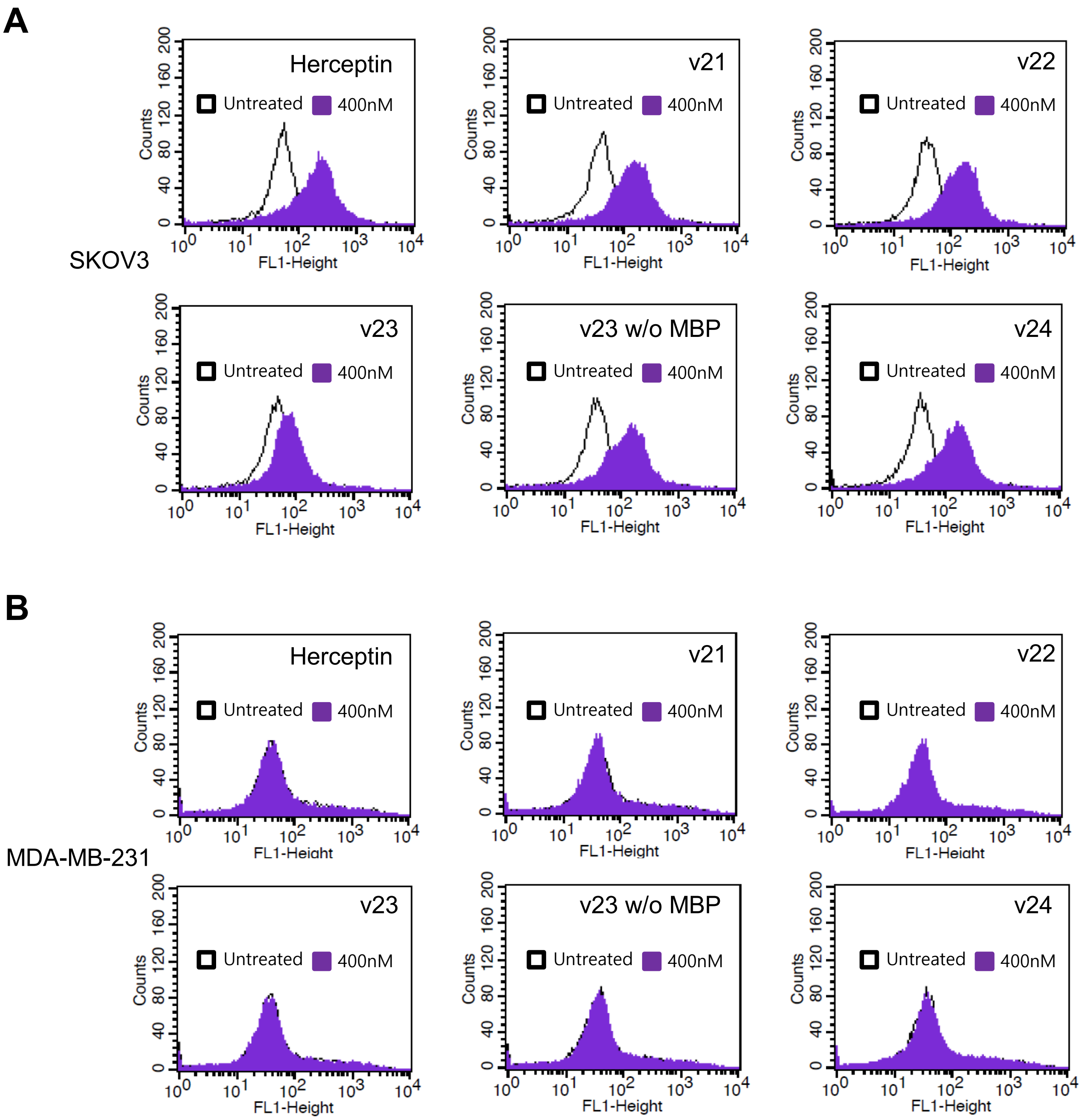

Supplementary Figure S1. Raw data histograms of FACS analysis for binding of trastuzumab (Herceptin®) and v21–v24 products to SKOV3 (A) and MDA-MB-231 (B) cells.

Supplementary Table S1

Supplementary Table S1. Amino acid sequences of recombinant proteins expressed by v21–v24 constructs (for v23 and v24, regions for final products after enzymatic cleavage are indicated in red).

Construct	Amino acid sequence expressed by the construct
**v21**	MDIQMTQSPSSLSASVGDRVTITCRASQDVNTAVAWYQQKPGKAPKLLIYSASFLYSGVPS<br>RFSGSRSGTDFTLTISSLQPEDFATYYCQQHYTTPPTFGQGTKVEIKRSGGGGSGGGGSG<br>GGGSEVQLVESGGGLVQPGGSLRLSCAASGFNIKDTYIHWVRQAPGKGLEWVARIYPTNG<br>YTRYADSVKGRFTISADTSKNTAYLQMNSLRAEDTAVYYCSRWGGDGFYAMDYWGQGTLV<br>TVSSASGSEQKLISEEDLHHHHHHTGGSTSELEFVD
**v22**	MKYLLPTAAAGLLLLAAQPAMAHMDIQMTQSPSSLSASVGDRVTITCRASQDVNTAVAWYQ<br>QKPGKAPKLLIYSASFLYSGVPSRFSGSRSGTDFTLTISSLQPEDFATYYCQQHYTTPPTFG<br>QGTKVEIKRSGGGGSGGGGSGGGGSEVQLVESGGGLVQPGGSLRLSCAASGFNIKDTYIH<br>WVRQAPGKGLEWVARIYPTNGYTRYADSVKGRFTISADTSKNTAYLQMNSLRAEDTAVYYC<br>SRWGGDGFYAMDYWGQGTLVTVSSASGSEQKLISEEDLHHHHHHTGGSTSELEFVD
**v23**	MKTEEGKLVIWINGDKGYNGLAEVGKKFEKDTGIKVTVEHPDKLEEKFPQVAATGDGPDIIF<br>WAHDRFGGYAQSGLLAEITPDKAFQDKLYPFTWDAVRYNGKLIAYPIAVEALSLIYNKDLLPN<br>PPKTWEEIPALDKELKAKGKSALMFNLQEPYFTWPLIAADGGYAFKYENGKYDIKDVGVDN<br>AGAKAGLTFLVDLIKNKHMNADTDYSIAEAAFNKGETAMTINGPWAWSNIDTSKVNYGVTVL<br>PTFKGQPSKPFVGVLSAGINAASPNKELAKEFLENYLLTDEGLEAVNKDKPLGAVALKSYEE<br>ELAKDPRIAATMENAQKGEIMPNIPQMSAFWYAVRTAVINAASGRQTVDEALKDAQTNSSS<br>GENLYFQGDIQMTQSPSSLSASVGDRVTITCRASQDVNTAVAWYQQKPGKAPKLLIYSASFL<br>YSGVPSRFSGSRSGTDFTLTISSLQPEDFATYYCQQHYTTPPTFGQGTKVEIKRSGGGGSG<br>GGGSGGGGSEVQLVESGGGLVQPGGSLRLSCAASGFNIKDTYIHWVRQAPGKGLEWVARI<br>YPTNGYTRYADSVKGRFTISADTSKNTAYLQMNSLRAEDTAVYYCSRWGGDGFYAMDYWG<br>QGTLVTVSSASGSEQKLISEEDLHHHHHHTGGSTSELEFVD
**v24**	MKTEEGKLVIWINGDKGYNGLAEVGKKFEKDTGIKVTVEHPDKLEEKFPQVAATGDGPDIIF<br>WAHDRFGGYAQSGLLAEITPDKAFQDKLYPFTWDAVRYNGKLIAYPIAVEALSLIYNKDLLPN<br>PPKTWEEIPALDKELKAKGKSALMFNLQEPYFTWPLIAADGGYAFKYENGKYDIKDVGVDN<br>AGAKAGLTFLVDLIKNKHMNADTDYSIAEAAFNKGETAMTINGPWAWSNIDTSKVNYGVTVL<br>PTFKGQPSKPFVGVLSAGINAASPNKELAKEFLENYLLTDEGLEAVNKDKPLGAVALKSYEE<br>ELAKDPRIAATMENAQKGEIMPNIPQMSAFWYAVRTAVINAASGRQTVDEALKDAQTNSSS<br>GESLFKGPRDYNPISSTICHLTNESDGHTTSLYGIGFGPFIITNKHLFRRNNGTLLVQSLHGV<br>FKVKNTTTLQQHLIDGRDMIIIRMPKDFPPFPQKLKFREPQREERICLVTTNFQTKSMSSMV<br>SDTSCTFPSSDGIFWKHWIQTKDGQCGSPLVSTRDGFIVGIHSASNFTNTNNYFTSVPKNF<br>MELLTNQEAQQWVSGWRLNADSVLWGGHKVFMVKPEEPFQPVKEATQLMNGSSSSGEN<br>LYFQGDIQMTQSPSSLSASVGDRVTITCRASQDVNTAVAWYQQKPGKAPKLLIYSASFLYSG<br>VPSRFSGSRSGTDFTLTISSLQPEDFATYYCQQHYTTPPTFGQGTKVEIKRSGGGGSGGGG<br>SGGGGSEVQLVESGGGLVQPGGSLRLSCAASGFNIKDTYIHWVRQAPGKGLEWVARIYPT<br>NGYTRYADSVKGRFTISADTSKNTAYLQMNSLRAEDTAVYYCSRWGGDGFYAMDYWGQG<br>TLVTVSSASGSEQKLISEEDLHHHHHHTGGSTSELEFVD

Original Gel Images used to generate Figure 2

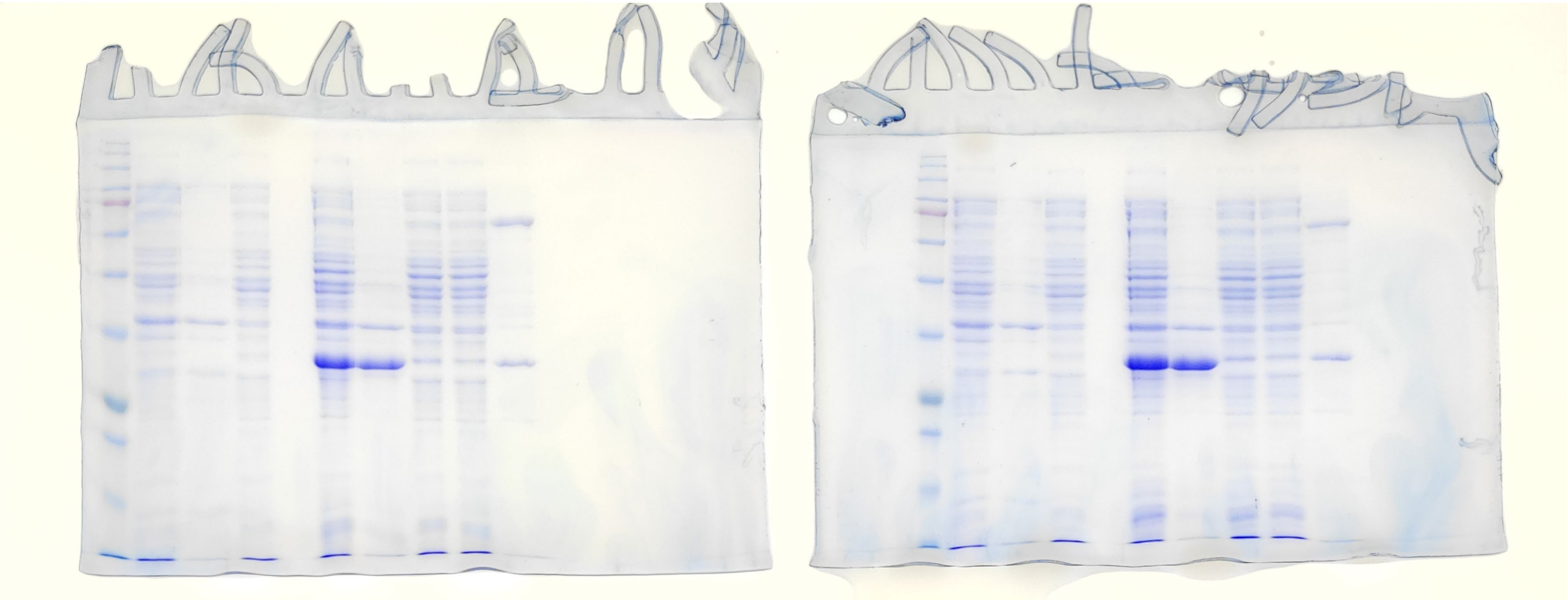

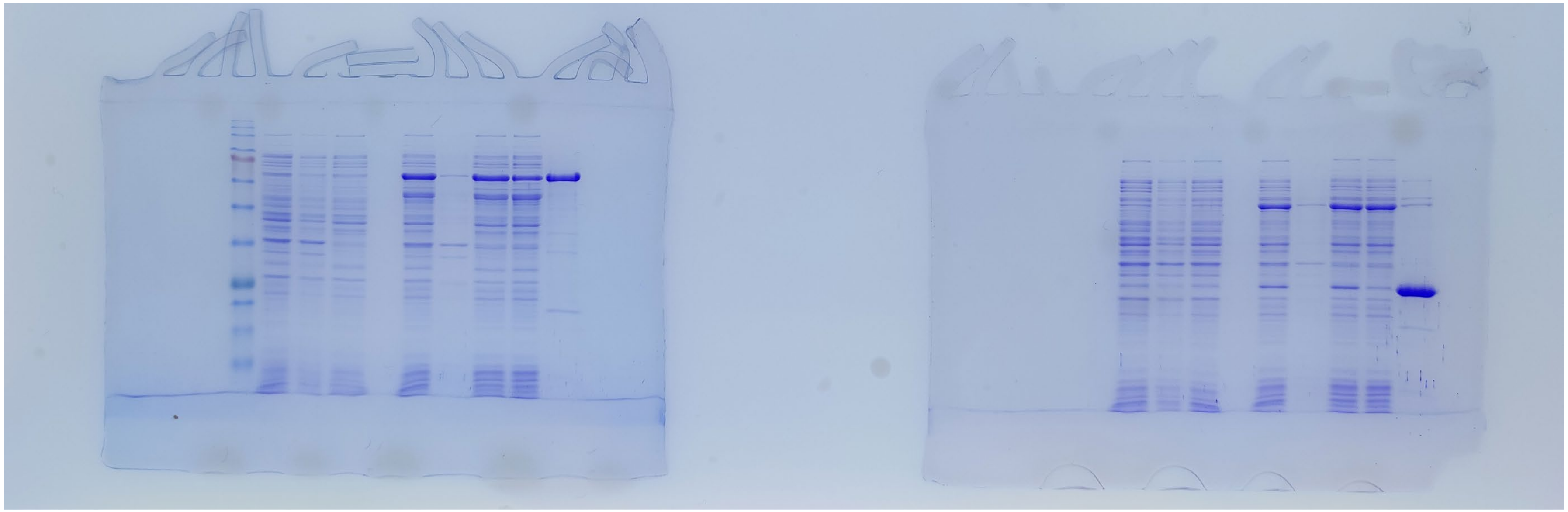

Original Gel Images used to generate Figure 3

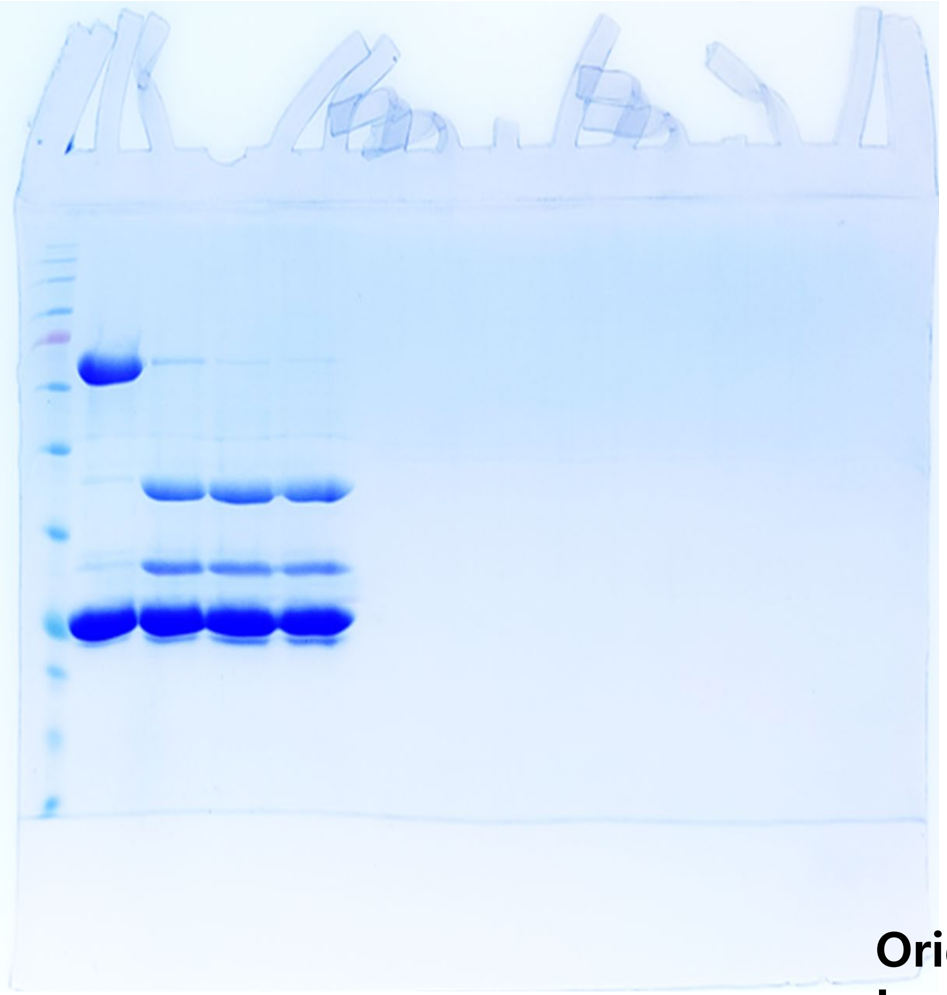

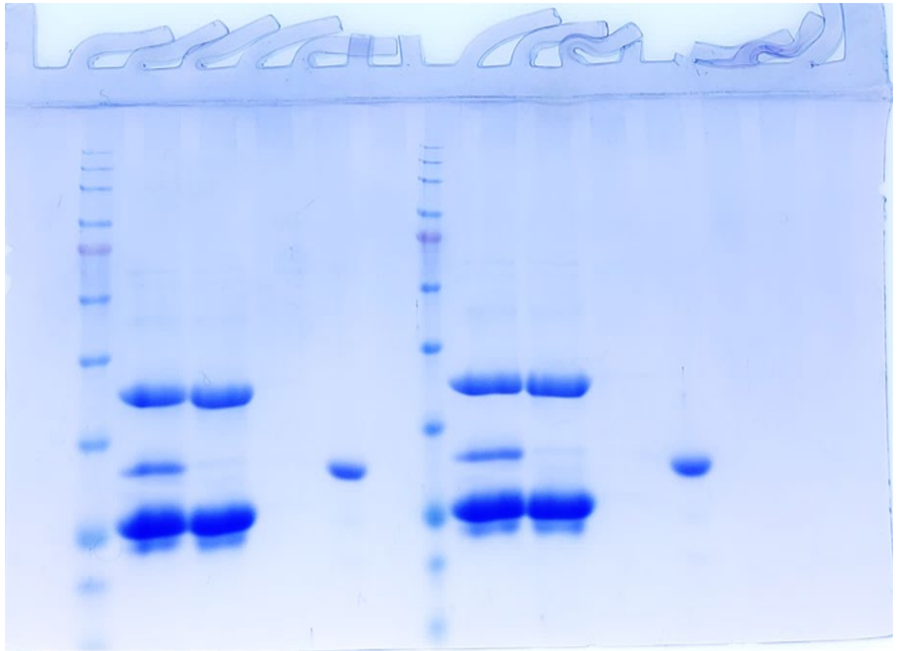

Original Gel Image used to generate Figure 5

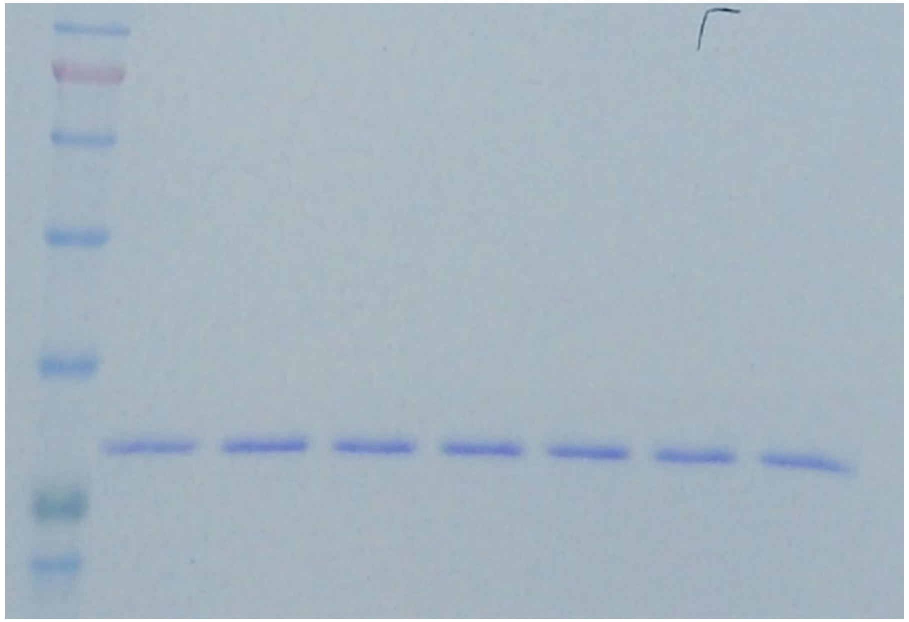

Supplement: Supplementary file 1 [file biomolecules-13-01508-s001.zip › biomolecules-2646540-supplementary.pdf]
